# Supplementary material for: Effects of summer schools: Who benefits the most?
Source: PLoS One. 2024 Apr 11;19(4):e0302060. doi: 10.1371/journal.pone.0302060 (PMC11008868; doi:10.1371/journal.pone.0302060)
Supplement: S1 Table — (PDF) [file pone.0302060.s001.pdf]

S1 Table. Description of the Dutch grading system.

**Dutch grade**	**American grade**	**Description**
9.5-10<br>8.5-9.0	A+<br>A	Excellent
8.0<br>7.5	A<br>B+	Very Good
7.0<br>6.5	B<br>C+	Good
6.0<br>5.5	C<br>D	Sufficient
5.0<br><5.0	F<br>F	Fail
