# Supplementary material for: Effects of summer schools: Who benefits the most?
Source: PLoS One. 2024 Apr 11;19(4):e0302060. doi: 10.1371/journal.pone.0302060 (PMC11008868; doi:10.1371/journal.pone.0302060)
Supplement: S2 Table — (PDF) [file pone.0302060.s002.pdf]

S2 Table. OLS regression of the DiD estimate for Z Math GPA: SES group classification based on 1 standard deviation minus/plus mean

	SES-1		SES-2		SES-3	
	Model 1	Model 2	Model 1	Model 2	Model 1	Model 2
Summer school	-1.106***	-1.016***	-1.165***	-1.130***	-1.138***	-1.028***
	(0.129)	(0.123)	(0.041)	(0.054)	(0.087)	(0.103)
Time indicator	-0.003	-0.00280	0.005	0.005	-0.047**	-0.047**
	(0.036)	(0.0360)	(0.022)	(0.022)	(0.0239)	(0.024)
Summer school * Time indicator	0.185	0.185	0.272***	0.272***	0.583***	0.583***
	(0.176)	(0.177)	(0.089)	(0.089)	(0.115)	(0.115)
Constant	-0.048	0.901**	0.053*	1.373***	0.057	1.029**
	(0.047)	(0.412)	(0.032)	(0.232)	(0.050)	(0.437)
Control variables		yes		yes		yes
Observations	4,640	4,640	21,422	21,422	5,576	5,576
Number of clusters	40	40	55	55	46	46
R-squared	0.029	0.067	0.018	0.067	0.013	0.080

* p < 0.10, ** p < 0.05, *** p < 0.01. Standard errors in parentheses are clustered at the school level

Note, included control variables are: gender, age, SES-group, grade level, education track, track advice, grade repetition, and year of participation
