# Supplementary material for: Effects of summer schools: Who benefits the most?
Source: PLoS One. 2024 Apr 11;19(4):e0302060. doi: 10.1371/journal.pone.0302060 (PMC11008868; doi:10.1371/journal.pone.0302060)
Supplement: S3 Table — (PDF) [file pone.0302060.s003.pdf]

S3 Table. OLS regression of the DiD estimate for Z Math GPA: SES group classification based on 0.5 standard deviation minus/plus mean

	SES-1		SES-2		SES-3	
	Model 1	Model 2	Model 1	Model 2	Model 1	Model 2
Summer school	-1.152***	-1.073***	-1.122***	-1.114***	-1.168***	-1.073***
	(0.092)	(0.083)	(0.055)	(0.063)	(0.075)	(0.100)
Time indicator	-0.003	-0.003	0.014	0.0142	-0.031	-0.031
	(0.027)	(0.027)	(0.025)	(0.025)	(0.024)	(0.024)
Summer school * Time indicator	0.217	0.217	0.241**	0.241**	0.493***	0.493***
	(0.157)	(0.157)	(0.097)	(0.097)	(0.100)	(0.100)
Constant	0.003	0.590**	0.053	1.660***	0.082**	1.181***
	(0.044)	(0.290)	(0.033)	(0.281)	(0.041)	(0.355)
Control variables		yes		yes		yes
Observations	7,776	7,776	13, 136	13,136	10,726	10,726
Number of clusters	50	50	55	55	49	49
R-squared	0.027	0.061	0.018	0.068	0.013	0.079

* p < 0.10, ** p < 0.05, *** p < 0.01. Standard errors in parentheses are clustered at the school level

Note, included control variables are: gender, age, SES-group, grade level, education track, track advice, grade repetition, and year of participation
