# Supplementary material for: Effects of summer schools: Who benefits the most?
Source: PLoS One. 2024 Apr 11;19(4):e0302060. doi: 10.1371/journal.pone.0302060 (PMC11008868; doi:10.1371/journal.pone.0302060)
Supplement: S4 Table — (PDF) [file pone.0302060.s004.pdf]

S4 Table. OLS regression of the DiD estimate for Z Math GPA: SES group classification based on 4 equal groups

	SES-1		SES-2		SES-3		SES4	
	Model 1	Model 2	Model 1	Model 2	Model 1	Model 2	Model 1	Model 2
Summer school	-1.154***	-1.071***	-1.287***	-1.288***	-0.975***	-0.912***	-1.139***	-1.051***
	(0.091)	(0.083)	(0.062)	(0.072)	(0.098)	(0.121)	(0.0560)	(0.092)
Time indicator	-0.003	-0.003	0.005	0.005	0.018	0.018	-0.044**	-0.044**
	(0.026)	(0.026)	(0.027)	(0.027)	(0.031)	(0.031)	(0.022)	(0.022)
Summer school * Time indicator	0.217	0.217	0.397***	0.397***	0.047	0.047	0.562***	0.562***
	(0.157)	(0.157)	(0.101)	(0.101)	(0.122)	(0.122)	(0.089)	(0.089)
Constant	0.006	0.572**	0.070*	1.978***	0.038	1.451***	0.051	0.829**
	(0.039)	(0.292)	(0.039)	(0.302)	(0.036)	(0.364)	(0.047)	(0.414)
Control variables		yes		yes		yes		yes
Observations	7,924	7,924	7,900	7,900	8,282	8,282	7,532	7,532
Number of clusters	50	50	53	53	54	54	46	46
R-squared	0.026	0.061	0.021	0.069	0.014	0.077	0.014	0.079

* p < 0.10, ** p < 0.05, *** p < 0.01. Standard errors in parentheses are clustered at the school level

Note, included control variables are: gender, age, SES-group, grade level, education track, track advice, grade repetition, and year of participation
